# Thieno[3,4-*d*][1,3]dithiole-2-thione

**DOI:** 10.1107/S1600536811044084

**Published:** 2011-10-29

**Authors:** Hua-wen Wen, Qi Fang

**Affiliations:** a School of Chemistry and Chemical Engineering, Shandong University, Jinan 250100, People’s Republic of China; b State Key Laboratory of Crystal Materials, Shandong University, Jinan 250100, People’s Republic of China

## Abstract

In the title compound, C_5_H_2_S_4_, the terminal monocyclic S atom deviates by 0.117 (1) Å from the mean plane of the other non-H atoms (r.m.s. deviation = 0.001 Å). All six C—S bonds and the central C—C bond in the rings are characterized by π-conjugated lengths, endowing the mol­ecule with high π-conjugation. In the crystal, the mol­ecules are parallel packed, forming columnar stacks along the *a* axis. Short inter­molecular S⋯S contacts [3.397 (1) and 3.486 (1) Å], are observed.

## Related literature

For details of the synthesis, see: Chiang *et al.* (1983 ▶); Gronowitz & Moses (1962 ▶). For DFT calculations using *GAUSSIAN*, see: Frisch *et al.* (2003 ▶). 
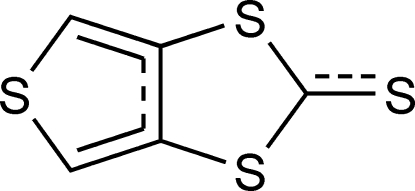


## Experimental

### 

#### Crystal data

C_5_H_2_S_4_
*M* *_r_* = 190.31
Orthorhombic, 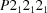

*a* = 3.9425 (1) Å
*b* = 9.2588 (2) Å
*c* = 19.2368 (3) Å
*V* = 702.20 (3) Å^3^
*Z* = 4
Mo *K*α radiation
μ = 1.25 mm^−1^
*T* = 294 K
0.20 × 0.06 × 0.03 mm

#### Data collection

Bruker APEX2 CCD area-detector diffractometer
Absorption correction: multi-scan (*SADABS*; Bruker, 2005 ▶) *T*_min_ = 0.792, *T*_max_ = 0.962
17824 measured reflections
1601 independent reflections
1486 reflections with *I* > 2σ(*I*)
*R*_int_ = 0.030

#### Refinement

*R*[*F* ^2^ > 2σ(*F* ^2^)] = 0.021
*wR*(*F* ^2^) = 0.053
*S* = 1.05
1601 reflections
90 parameters
All H-atom parameters refined
Δρ_max_ = 0.25 e Å^−3^
Δρ_min_ = −0.13 e Å^−3^
Absolute structure: Flack (1983 ▶), 618 Friedel pairs
Flack parameter: 0.18 (10)

### 

Data collection: *APEX2* (Bruker, 2005 ▶); cell refinement: *SAINT* (Bruker, 2005 ▶); data reduction: *SAINT*; program(s) used to solve structure: *SHELXS97* (Sheldrick, 2008 ▶); program(s) used to refine structure: *SHELXL97* (Sheldrick, 2008 ▶); molecular graphics: *SHELXTL* (Sheldrick, 2008 ▶); software used to prepare material for publication: *SHELXL97*.

## Supplementary Material

Crystal structure: contains datablock(s) I, global. DOI: 10.1107/S1600536811044084/bg2422sup1.cif

Structure factors: contains datablock(s) I. DOI: 10.1107/S1600536811044084/bg2422Isup2.hkl

Supplementary material file. DOI: 10.1107/S1600536811044084/bg2422Isup3.cml

Additional supplementary materials:  crystallographic information; 3D view; checkCIF report

## Figures and Tables

**Fig. 1.**
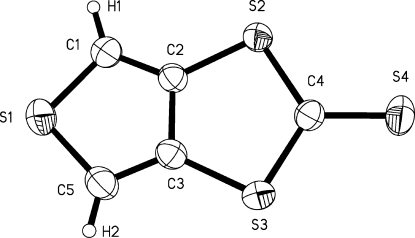
Molecular structure of the title compound. Displacement elliposoid is drawn at 50% probability level.

**Fig. 2.**
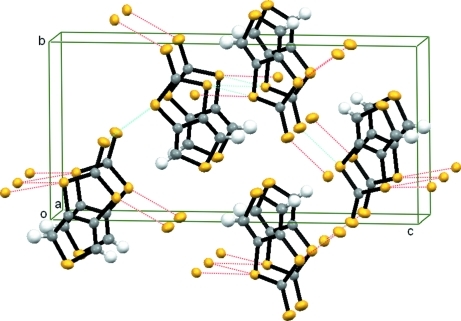
The packing of the molecules in title crystal viewed down the a axis, showing the column structure along this direction and S···S intermolecular short contacts between neighboring columns.

## supplementary crystallographic information

### Comment

Although the synthesis of the title compound was reported early by Gronowitz & Moses, 1962, there is no report about its crystal structure. We have recently re-synthesized this compound and determined its X-ray structure.

The molecule adopts a nearly planar C_2v_ conformation (see below for details). All the C—S bond lengths, from the longest 1.744 (2)Å to the shortest 1.635 (2) Å, are between the lengths of C—S single bond and C═S double bond. The central C≐C bond, shared by the two fused five-member rings, also features a delocalized bond with its length being 1.430 (2) Å. Thus the molecule is characterized by a highly planar π-conjugation.

The molecules pack in a paralleled way along the *a* axis, forming columnar stacks along this direction. The molecular packing is also characterized by many short intermolecular S···S contacts (for instance,  S2···S2 [-1/2 + *x*, 1/2 - *y*, -*z*] and S3···S4 [1 - *x*, -1/2 + *y*, 1/2 - *z*] distances are 3.397 (1)Å and 3.486 (1) Å, respectively). We believe that this kind of S···S intermolecular interactions helps to stabilitate the molecular packing in the crystal.

The terminal S4 atom deviates by 0.117 (1)Å from the least-squares plane through the other eight non-H atoms (C1, C2, C3, C4, C5, S1, S2, S3). We suppose that this deviation may be the result of the above S···S intermolecular interactions. To support this assumption, we carried out an optimization procedure for the molecular conformation by using the Gaussian-03 programs (Frisch *et al.*, 2003) within the framework of the DFT at the B3LYP/6–311(*d*) level. The optimized "free" molecule indeed adopts a perfect planar conformation with a strict C_2v_ symmetry. All the theoretical bond parameters are in good agreement with those of the X-ray results.

### Experimental

The title compound was synthesized by a similar procedure to Chiang *et al.* (1983). 3,4-Dibromothiophene (2.18 g, 9.0 mmol) was dissolved in 30 ml anhydrous diethyl ether and stirred in the presence of N_2_ at 195 K while *n*-butyllithium (5.6 ml, 9.0 mmol, 1.6 *M* in hexane) was added *via* syringe. Stirring was continued for 0.5 h, then sulfur (0.288 g, 9.0 mmol) was added. The reaction mixture was stirred for 1 h, and *n* -butyllithium (5.6 ml, 9.0 mmol) was added *via* syringe. After being stirred for 0.5 h, sulfur (0.288 g, 9.0 mmol) was added to the yellow solution. After 1 h, the reaction mixture was allowed to come to r.t. and was dried *in vacuo*. After removal of the solvent, 2 *M* sodium hydroxide solution (20 ml) and carbon disulfide (12 ml) were added. The mixture was refluxed under N_2_ at 363 K for 4 h. And then the solution was stirred overnight at r.t.. The excess of carbon disulfide was removed *in vacuo*. Filtration of the mixture gave a yellow solid. Recrytallization of the solid from dichloromethane-hexane (1:5, *v*/*v*) gave 0.26 g (15% yield) of the compound. Crystals were grown by slow evaporation of a dichloromethane solution.

### Refinement

Both H atoms were located in a difference Fourier map and freely refined in isotropic approximation, leading to C—H distances 0.88 (2), 0.94 (2) Å and Uiso 0.045 (5), 0.060 (7)Å^-1^.

### Crystal data

**Table:**

C_5_H_2_S_4_	*F*(000) = 384
*M**_r_* = 190.31	*D*_x_ = 1.800 Mg m^−^^3^
Orthorhombic, *P*2_1_2_1_2_1_	Mo *K*α radiation, λ = 0.71073 Å
Hall symbol: P 2ac 2ab	Cell parameters from 6079 reflections
*a* = 3.9425 (1) Å	θ = 2.4–26.6°
*b* = 9.2588 (2) Å	µ = 1.25 mm^−^^1^
*c* = 19.2368 (3) Å	*T* = 294 K
*V* = 702.20 (3) Å^3^	Bar, yellow
*Z* = 4	0.20 × 0.06 × 0.03 mm

### Data collection

**Table:**

Bruker APEX2 CCD area-detector diffractometer	1601 independent reflections
Radiation source: fine-focus sealed tube	1486 reflections with *I* > 2σ(*I*)
graphite	*R*_int_ = 0.030
Detector resolution: 8.3 pixels mm^-1^	θ_max_ = 27.5°, θ_min_ = 2.4°
phi and ω scans	*h* = −5→5
Absorption correction: multi-scan (*SADABS*; Bruker, 2005)	*k* = −12→12
*T*_min_ = 0.792, *T*_max_ = 0.962	*l* = −24→25
17824 measured reflections	

### Refinement

**Table:**

Refinement on *F*^2^	Secondary atom site location: difference Fourier map
Least-squares matrix: full	Hydrogen site location: difference Fourier map
*R*[*F*^2^ > 2σ(*F*^2^)] = 0.021	All H-atom parameters refined
*wR*(*F*^2^) = 0.053	*w* = 1/[σ^2^(*F*_o_^2^) + (0.0337*P*)^2^ + 0.0176*P*] where *P* = (*F*_o_^2^ + 2*F*_c_^2^)/3
*S* = 1.05	(Δ/σ)_max_ < 0.001
1601 reflections	Δρ_max_ = 0.25 e Å^−^^3^
90 parameters	Δρ_min_ = −0.13 e Å^−^^3^
0 restraints	Absolute structure: Flack (1983), 618 Friedel pairs
Primary atom site location: structure-invariant direct methods	Flack parameter: 0.18 (10)

### Special details

**Table:**

Experimental. Scan width 0.5° ω, Crystal to detector distance 5.96 cm
Geometry. All e.s.d.'s (except the e.s.d. in the dihedral angle between two l.s. planes)are estimated using the full covariance matrix. The cell e.s.d.'s are takeninto account individually in the estimation of e.s.d.'s in distances, anglesand torsion angles; correlations between e.s.d.'s in cell parameters are onlyused when they are defined by crystal symmetry. An approximate (isotropic)treatment of cell e.s.d.'s is used for estimating e.s.d.'s involving l.s. planes.
Refinement. Refinement of *F*^2^ against ALL reflections. The weighted *R*-factor *wR* and goodness of fit *S* are based on *F*^2^, conventional *R*-factors *R* are based on *F*, with *F* set to zero for negative *F*^2^. The threshold expression of *F*^2^ > σ(*F*^2^) is used only for calculating *R*-factors(gt) *etc*. and is not relevant to the choice of reflections for refinement. *R*-factors based on *F*^2^ are statistically about twice as large as those based on *F*, and *R*- factors based on ALL data will be even larger.

### Fractional atomic coordinates and isotropic or equivalent isotropic displacement parameters (Å^2^)

**Table:**

	*x*	*y*	*z*	*U*_iso_*/*U*_eq_	
S1	0.00458 (13)	−0.19316 (5)	0.07833 (3)	0.04533 (13)	
S2	−0.01295 (11)	0.26695 (5)	0.07145 (2)	0.03376 (11)	
S3	0.28209 (12)	0.16829 (5)	0.20312 (2)	0.03822 (12)	
S4	0.25260 (16)	0.48015 (5)	0.17223 (3)	0.05033 (14)	
C1	−0.0743 (5)	−0.0312 (2)	0.03940 (10)	0.0403 (4)	
C2	0.0144 (4)	0.08048 (18)	0.08177 (8)	0.0306 (3)	
C3	0.1522 (4)	0.03202 (19)	0.14643 (9)	0.0321 (3)	
C4	0.1774 (4)	0.31270 (18)	0.15017 (8)	0.0313 (4)	
C5	0.1610 (5)	−0.1139 (2)	0.15177 (11)	0.0415 (4)	
H1	−0.168 (5)	−0.030 (2)	−0.0057 (13)	0.060 (7)*	
H2	0.230 (5)	−0.162 (2)	0.1886 (10)	0.045 (5)*	

### Atomic displacement parameters (Å^2^)

**Table:**

	*U*^11^	*U*^22^	*U*^33^	*U*^12^	*U*^13^	*U*^23^
S1	0.0560 (3)	0.0293 (2)	0.0507 (3)	0.0022 (2)	−0.0044 (3)	−0.00404 (19)
S2	0.0428 (2)	0.0297 (2)	0.0288 (2)	0.0009 (2)	−0.00395 (19)	0.00159 (16)
S3	0.0476 (2)	0.0372 (2)	0.0299 (2)	0.0011 (2)	−0.00840 (19)	0.00048 (17)
S4	0.0692 (3)	0.0328 (2)	0.0490 (3)	−0.0045 (3)	−0.0055 (3)	−0.0084 (2)
C1	0.0485 (11)	0.0348 (9)	0.0377 (10)	0.0017 (9)	−0.0078 (8)	−0.0023 (8)
C2	0.0331 (8)	0.0292 (8)	0.0296 (8)	0.0024 (7)	0.0005 (8)	0.0003 (6)
C3	0.0336 (8)	0.0323 (9)	0.0303 (8)	0.0014 (7)	0.0015 (6)	0.0023 (7)
C4	0.0323 (8)	0.0327 (9)	0.0289 (8)	−0.0002 (7)	0.0016 (6)	−0.0017 (7)
C5	0.0527 (11)	0.0326 (10)	0.0391 (10)	0.0054 (8)	−0.0018 (8)	0.0027 (8)

### Geometric parameters (Å, °)

**Table:**

S1—C1	1.705 (2)	S4—C4	1.6345 (17)
S1—C5	1.707 (2)	C2—C1	1.362 (2)
S2—C2	1.7412 (17)	C2—C3	1.429 (2)
S2—C4	1.7422 (17)	C3—C5	1.355 (2)
S3—C4	1.7308 (17)	C5—H2	0.879 (19)
S3—C3	1.7445 (18)	C1—H1	0.94 (2)
			
C1—S1—C5	92.94 (9)	C3—C5—S1	110.88 (15)
C2—S2—C4	96.62 (8)	C3—C5—H2	125.0 (13)
C4—S3—C3	96.94 (8)	S1—C5—H2	124.0 (12)
C1—C2—C3	112.34 (16)	C2—C1—S1	110.97 (14)
C1—C2—S2	131.94 (14)	C2—C1—H1	129.9 (14)
C3—C2—S2	115.72 (12)	S1—C1—H1	119.2 (14)
C5—C3—C2	112.87 (17)	S4—C4—S3	122.45 (10)
C5—C3—S3	131.76 (15)	S4—C4—S2	122.31 (10)
C2—C3—S3	115.37 (13)	S3—C4—S2	115.24 (9)
			
C4—S2—C2—C1	−177.48 (19)	S3—C3—C5—S1	−178.73 (11)
C4—S2—C2—C3	3.09 (15)	C1—S1—C5—C3	0.08 (16)
C1—C2—C3—C5	−0.7 (2)	C3—C2—C1—S1	0.7 (2)
S2—C2—C3—C5	178.87 (14)	S2—C2—C1—S1	−178.73 (12)
C1—C2—C3—S3	178.54 (13)	C5—S1—C1—C2	−0.46 (16)
S2—C2—C3—S3	−1.92 (18)	C3—S3—C4—S4	−177.35 (11)
C4—S3—C3—C5	178.7 (2)	C3—S3—C4—S2	2.45 (10)
C4—S3—C3—C2	−0.32 (14)	C2—S2—C4—S4	176.50 (11)
C2—C3—C5—S1	0.3 (2)	C2—S2—C4—S3	−3.29 (11)
